# Meta-analysis of stereotactic hematoma removal and craniotomy hematoma removal in the treatment of hypertensive intracerebral hemorrhage in the elderly

**DOI:** 10.1097/MD.0000000000036533

**Published:** 2023-12-08

**Authors:** Chao Tang, Min Zhang, Wei Li

**Affiliations:** a Department of neurosurgery, Linping Campus, The Second Affiliated Hospital of Zhejiang University School of Medicine, Hangzhou, China.

**Keywords:** craniotomy hematoma, hypertensive cerebral hemorrhage, meta-analysis, stereotactic hematoma

## Abstract

**Background:**

A large number of clinical studies suggested that surgery might be a better choice than conservative treatment for treating hypertensive intracerebral hemorrhage in the middle-aged and elderly. Stereotactic puncture can reduce the mass effect caused by hematoma, reduce the intervention of body homeostasis, reduce brain tissue damage, and improve the prognosis of patients with cerebral hemorrhage. This meta-analysis aims to evaluate the efficacy of stereotactic puncture and craniotomy in elderly patients with hypertensive intracerebral hemorrhage.

**Methods:**

A search strategy was designed to search in databases, including PubMed, Embase, Cochrane Central Register of Controlled Trials, CNKI, Wanfang database and relevant references. Literature on the efficacy and safety of different surgical methods for hypertensive cerebral hemorrhage in the middle-aged and elderly were retrieved. The search time was until August 17, 2022. Keywords included “hypertensive intracerebral hemorrhage,” “stereotactic hematoma removal,” “craniotomy.” After the literature search, 2 researchers independently conducted literature screening, quality evaluation of included trials and data extraction. RevMan5.4 software was used to perform a Meta-analysis on the operation time, hospital stay, postoperative Glasgow Coma Scale (GCS) score, postoperative daily activity ability, postoperative complications and neurological prognosis scores included in the included studies.

**Results:**

A total of 1988 samples were included in 9 studies. 1022 patients underwent stereotactic hematoma removal, and 968 patients underwent craniotomy hematoma removal. The orientation group had more advantages in the length of hospital stay, postoperative disability, pulmonary infection, intracranial infection and digestive tract ulcer, and the difference was statistically significant *(P* < .05). In addition, the length of stay (*I²*= 83%) of the included articles had good homogeneity (*I²*< 50%), and there was no significant difference between the 2 groups in operation time, postoperative GCS score, postoperative daily activity ability, and neurological prognosis score (*P* > .05).

**Conclusion:**

The meta-analysis indicate that compared to craniotomy for hematoma removal, stereotactic hematoma removal can reduce the postoperative disability rate, intracranial infection rate, lung infection rate, and digestive tract ulcer rate in middle-aged and elderly patients with hypertensive intracerebral hemorrhage.

## 1. Introduction

Hypertensive intracerebral hemorrhage (HICH) is a common neurosurgical disease that seriously endangers the life safety of middle-aged and elderly patients and brings a heavy economic burden to families and the society.^[1]^ The pathogenesis of HICH is usually the rupture of intracranial arteries, veins and capillaries caused by hypertension, and the mechanical stress of hematoma on brain tissue is the most common cause.^[2]^ Studies have shown that HICH accounts for 50–70% of all spontaneous intracerebral hemorrhage (ICH), and its incidence rate and mortality rate rank first among all types of strokes. Furthermore, more than 30% of the survivors have different degrees of disability.^[3–5]^ With the aggravation of population aging, the incidence rate of HICH continues to rise. One study showed that HICH patients with hematoma volume > 50ml would face higher death and disability rates.^[6]^

Although the harm of HICH to middle-aged and elderly patients is well known, there has been no major breakthrough in its treatment scheme so far. At present, conservative treatment and surgery are still the main options for HICH treatment.^[7]^ For a long time, HICH has been treated with traditional conservative methods. However, the research results in recent years showed that it was related to the high mortality^[8]^ and high mortality of HICH patients. The surgical treatment of HICH could be roughly divided into craniotomy and minimally invasive surgery. A craniotomy is the primary surgical treatment for HICH. Although it can completely eliminate hematoma, it has adverse effects such as sizeable surgical trauma, general anesthesia, apparent brain tissue damage, high bleeding volume, long operation time, severe edema reaction, many complications, poor prognosis and poor efficacy.^[9,10]^

In recent years, minimally invasive neurosurgery techniques such as stereotactic puncture have been proven to be an alternative method for treating cerebral hemorrhage.^[11,12]^ Many large case studies have reported the effectiveness of stereotactic puncture in patients with cerebral hemorrhage.^[13,14]^ It is believed that stereotactic aspiration can reduce the mass effect caused by hematoma, reduce the intervention of body homeostasis, reduce brain tissue damage, and improve the prognosis of patients with cerebral hemorrhage.^[15]^ However, compared with craniotomy, the efficacy and safety of stereotactic aspiration in the treatment of hypertensive intracerebral hemorrhage in middle-aged and elderly patients are still uncertain. With the publication of literature related to stereotactic aspiration and craniotomy in recent years,^[15–18]^ it is possible to further compare the effects of stereotactic aspiration and craniotomy in the treatment of cerebral hemorrhage.

Therefore, this Meta-analysis aims to determine the efficacy and safety of stereotactic hematoma removal in middle-aged and elderly patients with hypertensive intracerebral hemorrhage compared with craniotomy.

## 2. Materials and Methods

### 2.1. Literature search

The search strategy was designed and the literature related to the efficacy and safety of different surgical methods for hypertensive cerebral hemorrhage in the middle-aged and elderly were retrieved from PubMed, Embase, Cochrane Central Register of controlled trials (CENTRAL), CNKI, Wanfang database and relevant references. The search time was until August 17, 2022. Keywords included “hypertensive intracerebral hemorrhage,” “stereotactic hematoma removal,” “craniotomy.” After the literature search, 2 researchers independently conducted literature screening, quality evaluation of included trials and data extraction. RevMan5.4 software performed a Meta-analysis on the operation time, hospital stay, postoperative Glasgow Coma Scale (GCS) score, postoperative daily activity ability, postoperative complications and neurological prognosis scores included in the included studies. The search formulars of databases, mainly PubMed, Embase and CENTRAL, are shown in the supplementary materials, http://links.lww.com/MD/L31.

### 2.2. Inclusion and exclusion criteria

Inclusion criteria: Study type: whether it is a randomized controlled trial (RCT) or not, it can be included; subjects: patients were diagnosed with hypertensive cerebral hemorrhage; intervention group: stereotactic hematoma removal; Control group: craniotomy hematoma removal; study results of patients with complete age information and age > 45 years or separable age group > 45 years; preoperative blood loss or GCS score data were complete; Exclusion criteria: studies with a sample size of fewer than 10 cases, studies without access to full text and statistical methods; repeatedly published literature; non-core Chinese literature of Peking University. All included studies were ensured to follow consistent standards of inclusion and exclusion

### 2.3. Data extraction and quantity evaluation

Two investigators screened the included literature for data, and disagreements were resolved through discussion. Information extracted included: first author, time of publication, sample size by subgroup, age, preoperative bleeding or GCS score, preoperative blood pressure values or history of hypertension, and expected study outcome.

The quality of the included RCTs was assessed using the Cochrane Risk of Bias Assessment Tool, and the risk of bias was evaluated in 6 aspects: random assignment method, allocation concealment scheme, blinding, completeness of outcome data, choice of reporting study results, and other biases. The risk of bias of the included RCTs was classified as “low risk,” “high risk,” and “unclear risk.” A total score < 2 was considered as low-quality literature. Non-RCT studies were assessed for quality using the Newcastle-Ottawa scale, which evaluated study quality in 4 aspects: sample selection, sample comparability, study outcome and follow-up, where follow-up time ≧ 6 months was considered to be the length of follow-up that could meet the requirements of clinical research, and a total score > 5 was considered to be high-quality literature.

### 2.4. Statistical methods

Meta-analysis was performed using RevMan 5.4 software, and *P* < .05 indicated that the differences were statistically significant. Risk ratio (RR) values and their 95% confidence interval (CI) were used to analyze dichotomous variables. Furthermore, STD Mean Difference (SDM) and the 95% CI were used to analyze continuous variables, and heterogeneity was tested by I^2^.

If *P* > .05, I^2^ < 50%, it indicates no obvious heterogeneity in the included studies, and the fixed effect model can be used. If *P* < .05, I^2^ > 50%, it indicates that the heterogeneity among the included studies is large, and then the random effect model is used. In addition, the source of heterogeneity is explored through sensitivity analysis by eliminating the included studies one by one and then combining the effect quantities. Publication bias was assessed using funnel plots. *P *≤ .05 was considered statistically significant.

## 3. Results

### 3.1. Literature search results

**Figure 1.**
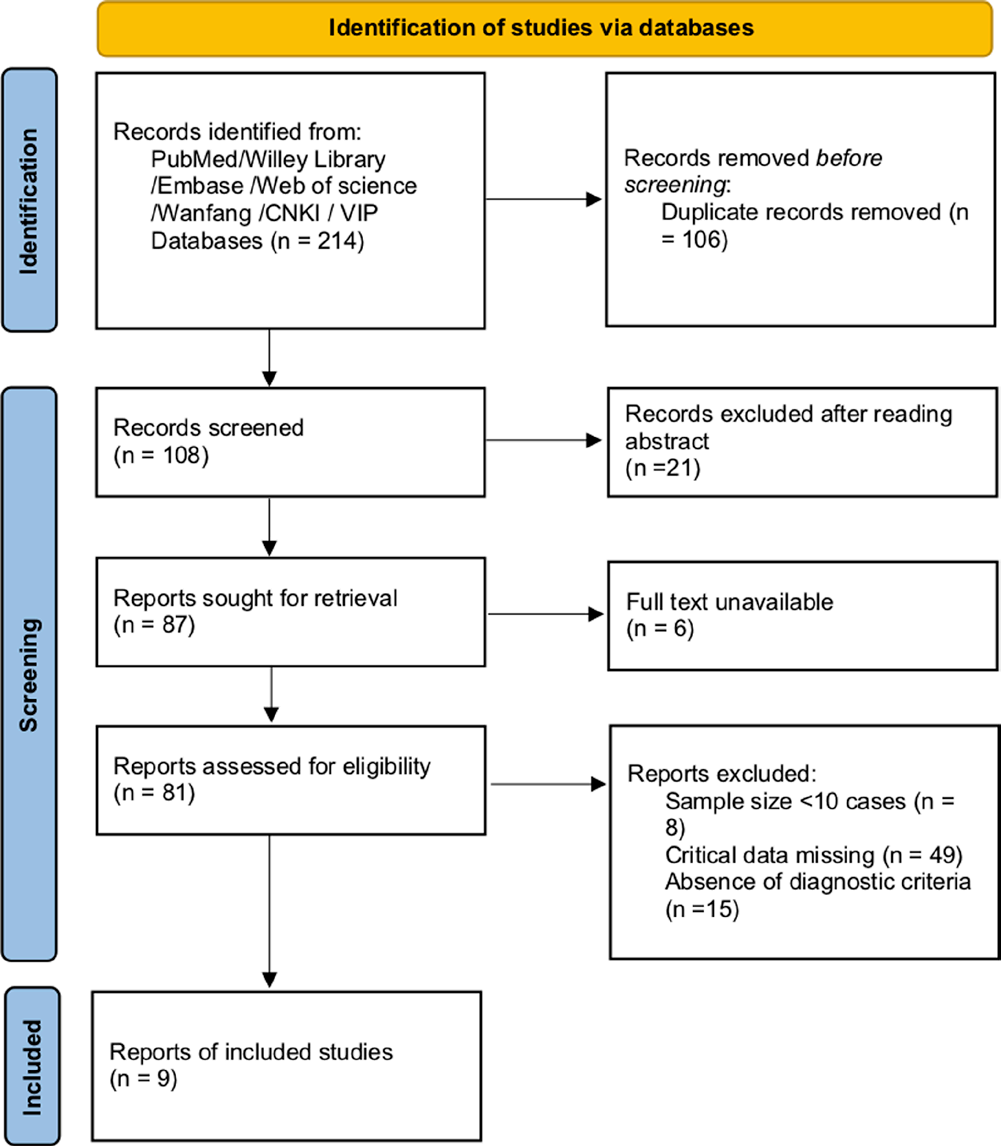
PRISAM flow diagram.

A total of 214 literature were obtained from the database and reference search. A total of 106 duplicate articles were removed, and 21 were removed after reading the abstract. There are still 6 articles that cannot be obtained in full text. After the full-text screening, 9 studies were finally included. The screening process is shown in Figure 1.

### 3.2. Basic information included in the study

The study compared the basic information of the included studies and the baseline information of the patients with hypertensive intracerebral hemorrhage > 45 years old before undergoing stereotactic hematoma removal or craniotomy. The 9 articles included 1 RCT study^[16]^ and 8 retrospective chart reviews (RCR).^[15,17–23]^ A total of 1988 patients were included in the study. Among them, 1022 patients underwent stereotactic hematoma removal, and 968 patients underwent craniotomy hematoma removal. There was no significant difference in the baseline characteristics of the included patients. The age of the patients was > 45 years. All the patients included in the study had a local diagnosis of a cerebral hemorrhage at baseline and had preoperative systolic blood pressure ≥ 140 mmHg, diastolic blood pressure ≥ 90 mmHg or a history of hypertension. Among them, 76.9% of the patients in the stereotactic and craniotomy groups in Guo, 2022 study met the diagnostic criteria for hypertension. However, due to the relatively new publication time and high research quality (Newcastle Ottawa scale score = 7 points), after discussion by 2 researchers, they decided to be included in the study and carried out a sensitivity analysis on the research results related to the neurological function prognosis score (mRS). The follow-up time of 5 studies is ≥ 6 months, the follow-up time of 2 studies is 3 months, and the follow-up time of 2 studies is 2 months. The difference in follow-up time will be reflected in the literature quality evaluation and sensitivity analysis results. The basic characteristics of the included documents are shown in Table 1

**Table 1:** Basic information of the included literature.

Authors	Study design	Cases		Age (yr)		Bleeding volume (ml)		GCS score (points)		Blood pressure (mm Hg)		Observation index	Follow-up period (mo)
		Control group	Craniotomy group	Control group	Craniotomy group	Control group	Craniotomy group	Control group	Craniotomy group	Control group	Craniotomy group		
Guo, 2020 ^[15]^	Non-RCT	304	107	<60: 178 ≧60: 126	<60: 65 ≧60: 42	>20–<40: 161 ≥40–<80: 253 ≥80: 102	>20–<40: 12 ≥40–<80: 50 ≥80: 45	-	-	-	-	⑥	6
Zhou, 2011 ^[16]^	RCT	90	78	57.6 ± 11.2	59.2 ± 10.7	-	-	8.1 ± 2.3	8.4 ± 3.2	SBP:174.5 ± 13.2 DBP:102.2 ± 8.3	SBP: 172.9 ± 11.5 DBP:99.2 ± 7.6	③④⑤⑥	6
Wang, 2019 ^[17]^	Non-RCT	30	30	53.1 ± 5.6	54.8 ± 4.7	44.28 ± 1.43	43.57 ± 3.29	11.56 ± 1.46	11.79 ± 1.36	184.36 ± 23.67	190.27 ± 20.37	①②④⑤	3
Wei, 2016 ^[18]^	Non-RCT	42	45	69.7 ± 6.0	68.1 ± 5.4	46.4 ± 9.3	44.8 ± 6.4	7.9 ± 1.2	8.0 ± 1.1	>149.63/94.80	>149.63/94.8	①②③	6
Geng, 2015^[19]^	Non-RCT	12	28	60.92 ± 7.98	57.33 ± 6.73	17.64 ± 3.33	19.41 ± 3.74	8.92 ± 2.43	9.22 ± 2.21	-	-	①⑤	3
Ma, 2014^[20]^	Non-RCT	28	27	70.50 ± 17.30	65.20 ± 16.80	23.20 ± 6.80	21.30 ± 7.20	-	-	-	-	①④	2
Li, 2018 ^[21]^	Non-RCT	23	27	62.50 ± 7.30	60.20 ± 8.10	15.50 ± 3.60	14.30 ± 3.40	11.60 ± 2.90	12.10 ± 1.80	SBP: 168.6 _x0007_ 13.9	SBP: 172.3 _x0007_ 16.9	⑤	12
Yang, 2017^[22]^	Non-RCT	16	16	58.30 ± 2.70	60.10 ± 1.40	23.30 ± 2.20	22.40 ± 2.30	-	-	-	-	⑤④	2
Chi, 2014 ^[23]^	Non-RCT	C1:306	A:312	57.3 ± 10.1	63.4 ± 8.62	61.5 ± 23.8	83.4 ± 27.51	-	-	-	-	④	6
		C2:169	B:298	63.3 ± 8.63	62.7 ± 9.30	37.4 ± 20.62	56.3 ± 19.31	-	-	-	-		

Observation index ①: Operation time; ②: Hospital stay; ③: postoperative GCS score; ④: postoperative daily activity (ADL); ⑤ Postoperative complications (including postoperative rebleeding, intracranial infection, pulmonary infection and digestive tract ulcer); ⑥: neurological prognosis score (mRS).

### 3.3. Quality evaluation of included studies

**Figure 2.**
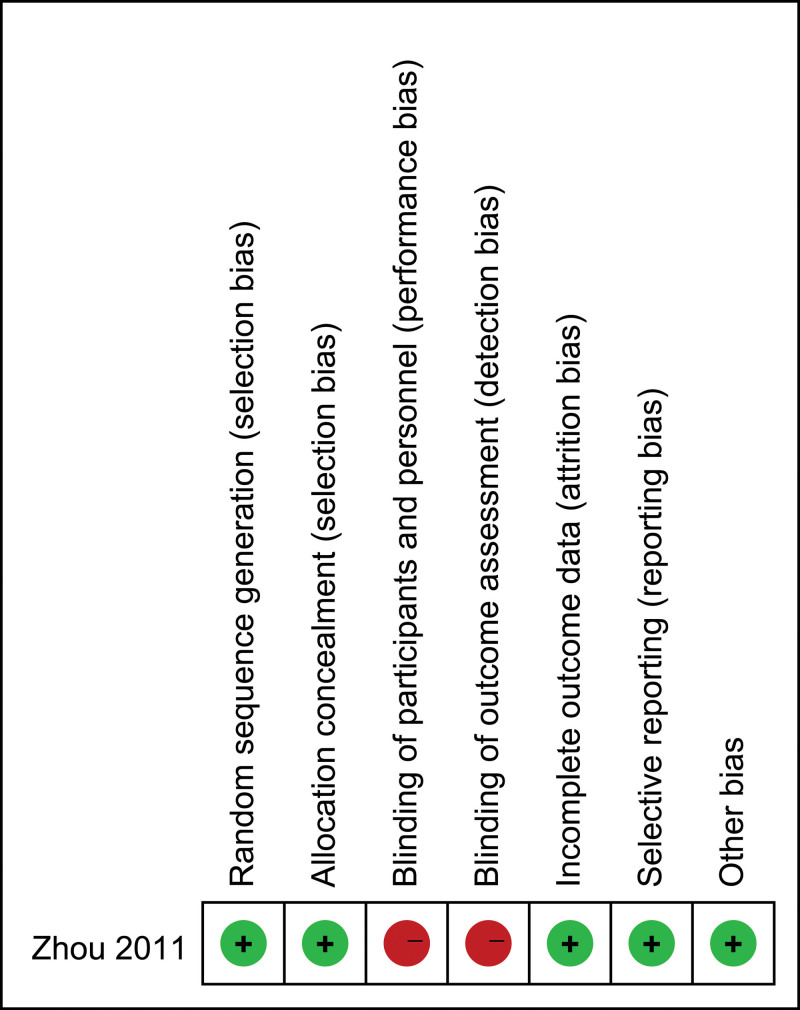
Quality evaluation of included RCT studies.

According to the results of the Cochrane bias risk assessment tool and Newcastle Ottawa scale, 9 studies^[15–23]^ were all high-quality studies, and the quality evaluation scores of 4 non-RCT studies^[17,19,20,22]^ were relatively low because the follow-up time was <6 months. The results of the bias risk assessment are shown in Figure 2, and the quality assessment results are shown in Table 2.

**Table 2:** Quality evaluation of included NON-RCT studies.

Authors	Score
Guo,2020^[15]^	7
Wang, 2019^[17]^	6
Wei, 2016^[18]^	8
Geng, 2015^[19]^	6
Ma, 2014^[20]^	6
Li, 2018^[21]^	7
Yang, 2017^[22]^	6
Chi, 2014^[23]^	8

### 3.4. Operation time

**Figure 3.**
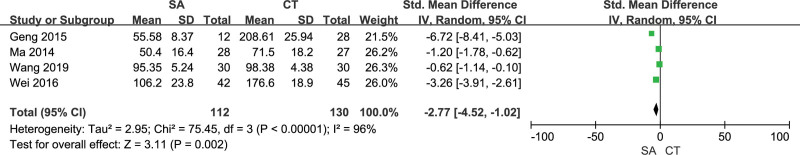
Forest chart: comparison of operation time between 2 group.

**Figure 4.**
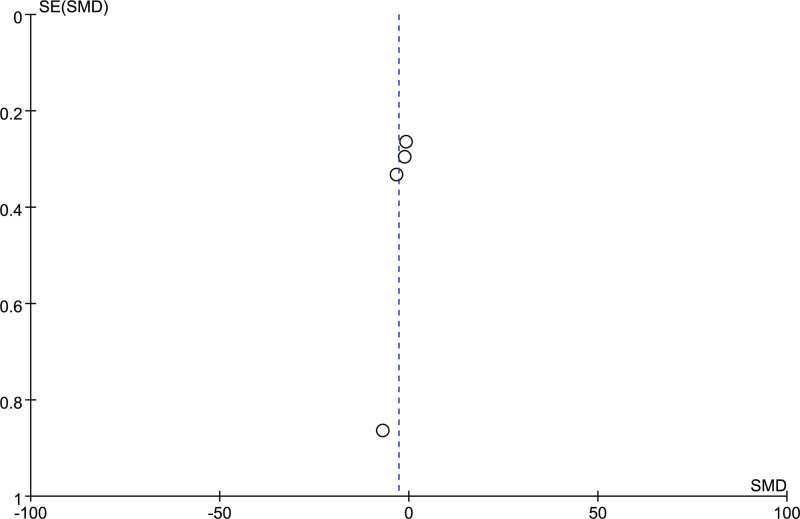
Funnel chart: comparison of operation time between 2 groups.

**Figure 5.**
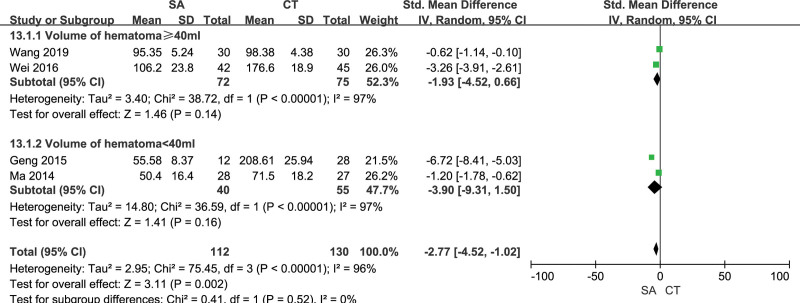
Forest chart: effect of preoperative blood loss on operation time.

4 studies included the comparison of the operation time between the 2 groups, including 112 patients in the stereotactic group and 130 patients in the craniotomy group. The included studies were heterogeneous (*I²*= 96%), using the random effect model, the operation time of the orienting group was shorter than that of the craniotomy group, and the difference was statistically significant [SMD = −2.27, 95% CI [−4.52, −1.02], *P* = .002] (Fig. 3). The funnel plot showed the scatter to the right with publication bias (Fig. 4). Subgroup analysis was performed according to the preoperative bleeding volume to find the source of heterogeneity. There was no significant difference between the preoperative bleeding volume ≥ 40ml subgroup and the preoperative bleeding volume < 40ml subgroup [SMD = −1.93, 95% CI [−4.52, 0.66], *P* = .14; SMD = −3.9, 95%CI [−9.31, 1.50], *P* = .16). There was still heterogeneity (I^2^ = 97%) among the studies in subgroup 2, but there was no heterogeneity (I^2^ = 0%) between subgroup 2 (Fig. 5).

### 3.5. Comparison of postoperative disability

**Figure 6.**
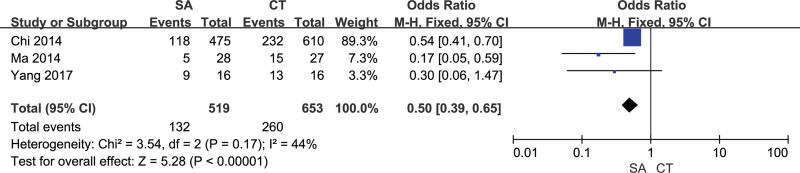
Forest chart: comparison of postoperative disability of 2 groups of patient.

**Figure 7.**
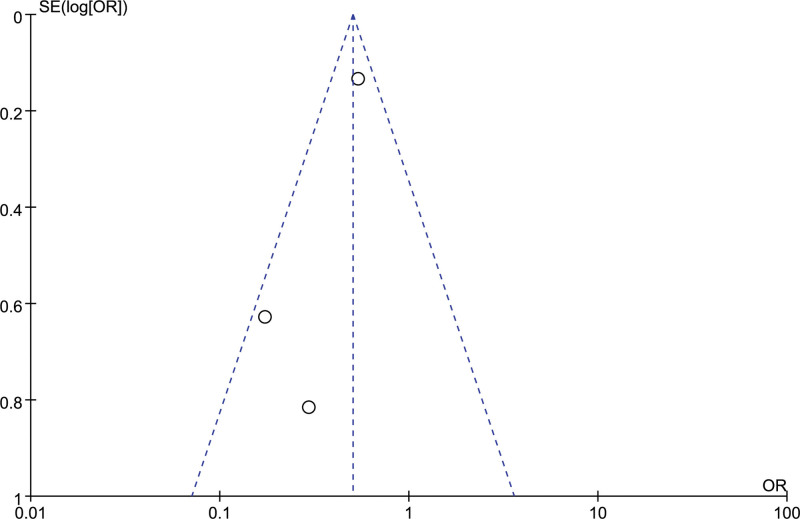
Funnel chart: comparison of postoperative disability of patients in 2 groups.

Postoperative daily living ability and disability were assessed using the activities of daily living (ADL) scale. Patients with an ADL rating of < 60 were considered as postoperative disabilities. 3 studies evaluated the number of patients with postoperative disability in the 2 groups, and there was no heterogeneity between the studies (*I²*= 44%). The number of postoperative disabled patients in the fixed effect model orientation group was lower than that in the craniotomy group, and the difference was statistically significant [OR = 0.5,95% CI [0.39, 0.56], *P *< .05] (Fig. 6). Funnel scatter points were distributed within the 95% CI line, suggesting no publication bias between studies (Fig. 7). The sensitivity analysis results indicated that the sensitivity of the analysis results was low, and the difference of excluding any study was statistically significant (*P *≤ .05).

### 3.6. Postoperative rebleeding

**Figure 8.**
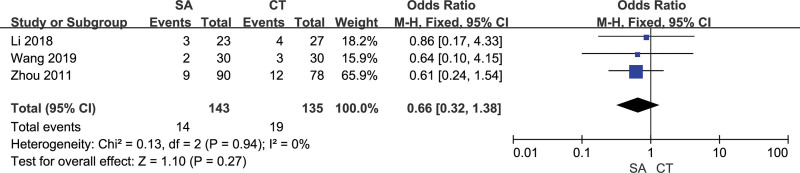
Forest chart: comparison of postoperative bleeding in 2 groups.

**Figure 9.**
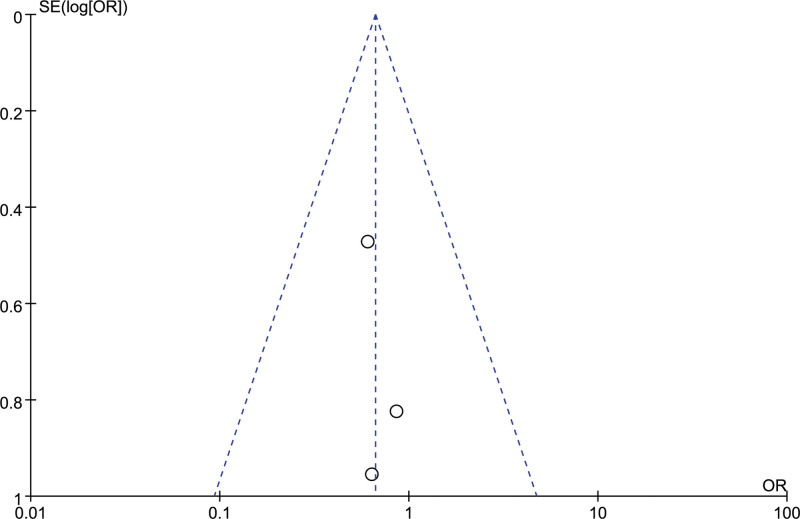
Funnel chart: comparison of postoperative bleeding in 2 groups.

3 studies evaluated the postoperative rebleeding of the 2 groups of patients, with good homogeneity between the studies (*I²*= 0%). The fixed effect model was used and observed no significant difference in postoperative rebleeding between the orienting group and the craniotomy group [OR = 0.66, 95% CI [0.32, 1.38], *P* = .27] (Fig. 8). Funnel scatter points were all distributed within the 95%CI line, and there was no publication bias (Fig. 9).

### 3.7. Intracranial infection

**Figure 10.**
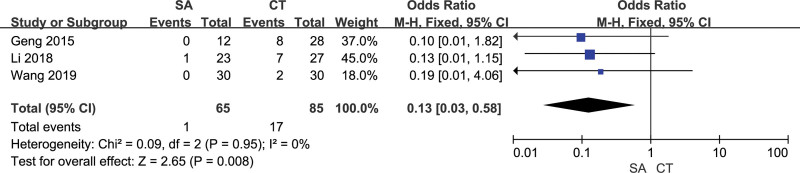
Forest chart: comparison of postoperative intracranial infection in 2 groups.

**Figure 11.**
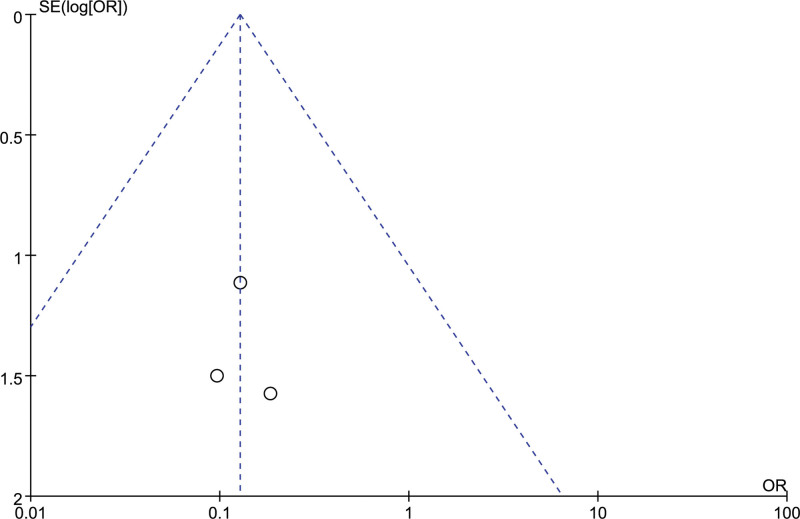
Funnel chart: comparison of postoperative intracranial infection in 2 groups.

3 studies evaluated the postoperative intracranial infection of patients, and there was no heterogeneity between included studies (*I²*= 0%). The results of the fixed effect model analysis showed that the intracranial infection in the orienting group was better than that in the craniotomy group, and the difference was statistically significant [OR = 0.13, 95% CI [0.03, 0.58], *P *= .008] (Fig. 10). The sensitivity analysis results showed that this analysis results were relatively stable, and the differences of any 1 study were statistically significant (*P* ≤ .05). Funnel plots suggested that the included studies had no publication bias (Fig. 11).

### 3.8. Pulmonary infection

**Figure 12.**
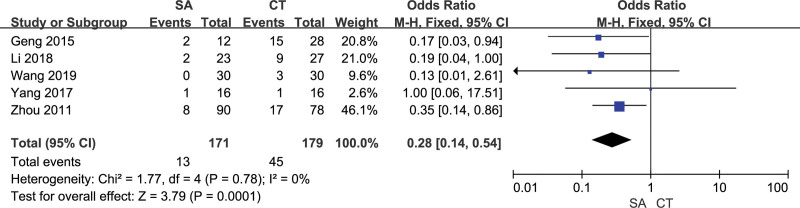
Forest chart: comparison of postoperative pulmonary infection in 2 groups.

**Figure 13.**
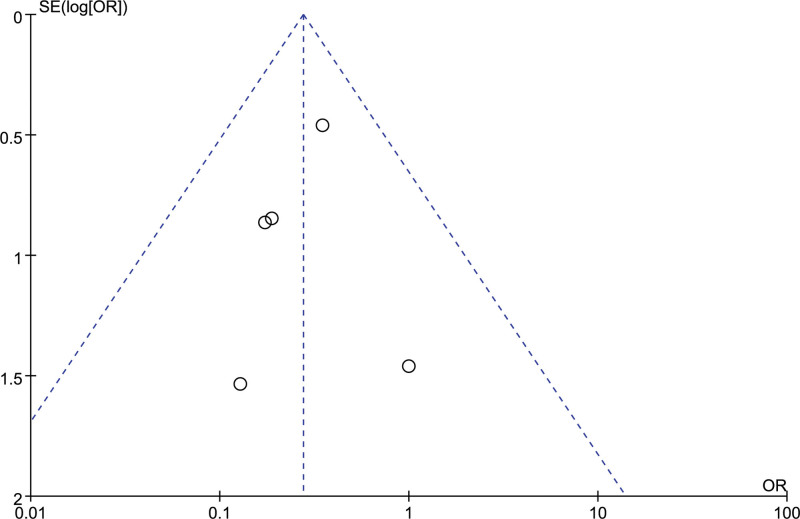
Funnel chart: comparison of postoperative pulmonary infection in 2 groups.

5 studies evaluated the postoperative intracranial infection of patients, 171 in the orientation group and 179 in the craniotomy group. There was no heterogeneity among the included studies (*I²*= 0%). The results of the fixed effect model analysis showed that the postoperative pulmonary infection in the orienting group was better than that in the craniotomy group, and the difference was statistically significant [OR = 0.28, 95% CI [0.14, 0.54], *P* = .0001] (Fig. 12). The sensitivity analysis results showed that the results were relatively stable, and the differences of any 1 study were statistically significant (*P* ≤ .05). The funnel plot suggested no publication bias in the included studies (Fig. 13).

### 3.9. Digestive tract ulcer

**Figure 14.**
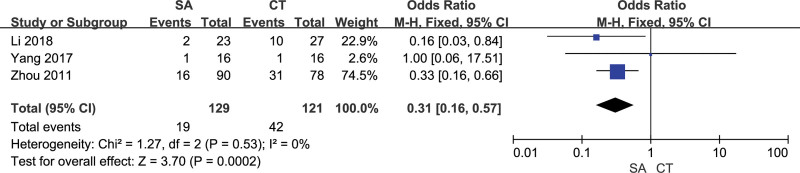
Forest chart: comparison of postoperative digestive tract ulcer in 2 groups.

**Figure 15.**
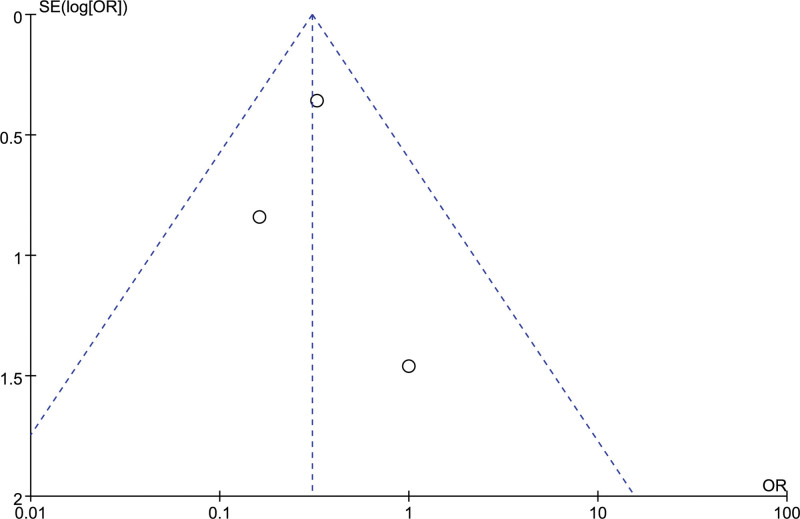
Funnel chart: comparison of postoperative digestive tract ulcer in 2 groups.

3 studies evaluated the postoperative digestive tract ulcer of patients, and there was no heterogeneity between included studies (*I²*= 0%). The fixed effect model analysis results showed that the digestive tract ulcer in the orientation group was better than that in the craniotomy group. The difference was statistically significant [OR = 0.31, 95% CI [0.16, 0.57], *P* = .0002] (Fig. 14). The sensitivity analysis showed that the results were relatively stable. The differences in all studies were statistically significant (*P *≤ .05). Funnel plots suggested that the included studies had no publication bias (Fig. 15).

## 4. Discussion

In this Meta-analysis, the results of 9 randomized controlled and retrospective medical record reviews were combined to study the efficacy and safety of stereotactic hematoma removal in the treatment of middle-aged and elderly patients with hypertensive intracerebral hemorrhage compared with craniotomy. The efficacy of the 2 surgical methods was evaluated by the evaluation results of the operation time and postoperative disability rate. The safety of the 2 surgical methods was comprehensively evaluated by the number of postoperative rebleeding, intracranial infection, pulmonary infection and digestive tract ulcers. The main results were as follows: (1) The orienting group had shorter operation and hospitalization time than the craniotomy group; However, the subgroup analysis based on the preoperative bleeding volume showed that there was no significant difference in the operation time among the subgroups when the preoperative bleeding volume of 40ml was used as the boundary; (2) Compared with the craniotomy group, the orienting group had better postoperative daily activity and lower disability rate, but there was no significant difference in GCS score^[24]^ and mRS score; (3) The postoperative intracranial infection, pulmonary infection and digestive tract ulcer in the orienting group were better than those in the craniotomy group. There was no significant difference between the 2 groups in postoperative rebleeding.

The operation duration may be related to the amount of preoperative bleeding and the size of the surgical wound. The stereotactic surgical catheterization can be achieved by drilling or direct puncture on the skull. The scalp incision with a diameter of 4cm in craniotomy maybe 3–4 times the area of the stereotactic puncture wound.^[25]^ Stereotactic hematoma removal does not require bone flap craniotomy. The residual hematoma was vacuumed out of the brain, and the residual hematoma was removed more thoroughly after the operation with urokinase dissolution. The intracranial hematoma was resected by multi-orbital and multi-target approaches. This surgical method can accurately locate the hematoma and help the surgeon to avoid important brain functional areas while accurately locating the target.^[26]^ At the same time, the progress of surgical robot technology and clinical practice have also been confirmed to improve the accuracy of stereotactic hematoma positioning further and shorten the operation time. Recent studies have shown that MRI-guided robots can complete hematoma removal at a speed of 9 minutes with an aiming accuracy of 1.26 ± 1.22 mm.^[27,28]^ It is worth noting that the included studies in the 2 subgroups with similar preoperative bleeding volumes showed no statistically significant difference in the operation time. The current studies retrieved by the researchers did not discuss the operation time or operation type selection for the preoperative bleeding volume of hypertensive cerebral hemorrhage patients. Further clinical studies are still needed to confirm the authenticity of this conclusion. However, suppose the results of this meta-analysis are credible. In that case, the possibility of publication bias should be considered regarding the influence of preoperative bleeding volume on the difference between stereotactic and craniotomy operation time.

The current study found that patients who underwent stereotactic hematoma removal had higher ADL scores. Although the therapeutic effect of craniotomy is undeniable, it is easy to damage the nerve during the operation. Common complications after craniotomy include neurological dysfunction, such as hemiplegic aphasia, mental disorder, and agnosia, which affect the life and work of patients.^[29]^ Therefore, compared with death, this paper pays more attention to whether stereotactic surgery and craniotomy can improve the quality of life of survival patients after surgery. The ADL score is an important basis for judging the prognosis of surgery for the following reasons. First, in acute diseases such as cerebral hemorrhage, postoperative dependence on others is a clinical result that is vital to the patient or directly related to the long-term quality of life of the patient after surgery. Many studies have reported data on death or dependence. Secondly, the current clinical decision-making of doctors pays more attention to patients’ quality of life after surgery. Simple survival or death data cannot answer this question. Finally, bias may occur when the trial collects data on many physiological endpoints due to the selective publication of endpoints showing significant therapeutic effects.^[30]^ Previous meta-analysis and clinical studies have also proposed that compared with craniotomy, the disability rate and dependency of the stereotactic puncture treatment group have decreased,^[31]^ which is the same as the results of this meta-analysis.

Given the problem that traditional craniotomy is easy to cause postoperative complications, this meta-analysis analyzed the situation of intracranial infection, pulmonary infection and digestive tract ulcer after operation to assist doctors in clinical decision-making. Stereotactic puncture uses a special silicone hose to minimize the damage to brain tissue, thus reducing the damage to nerve function. This study also showed that the incidence of postoperative complications in patients undergoing stereotactic hematoma removal was lower than that in patients undergoing craniotomy. Previous studies have shown that postoperative rebleeding can significantly increase the risk of poor prognosis.^[22]^ Although the results of this meta-analysis showed that the risk of postoperative rebleeding between the 2 groups was not statistically significant. While some included studies suggested that the number of postoperative rebleeding patients in the orientation group was lower than that in the craniotomy group. A previous meta-analysis also confirmed this conclusion.^[31]^ At the same time, the adverse prognosis may also be related to the operation timing, bleeding volume and bleeding site after bleeding.^[23]^ Most of the included studies lack individual patients’ data, so it is impossible to make a subgroup analysis on the influence of single factors on adverse reactions.

This study has some limitations. First, although we tried to include all the relevant studies, only 1 RCT study was included and the other studies were retrospective medical record reviews. Considering the quality of the electronic medical record filling in the hospital, the reliability of the results of such studies was lower than that of RCT studies. A total of 1988 participants were included in the 9 trials, but some unpublished or excluded studies due to non-PKU core might be omitted. Secondly, in the 2022 study by Guo, the proportion of hypertensive patients in the stereotactic group and craniotomy group was not 100%, which was a potential factor affecting the prognosis of patients. To exclude the risk of bias included in the Guo study, all variables involved in the Guo study were subjected to sensitivity analysis. The sensitivity analysis showed that the results of the meta-analysis included or excluded from this study were unchanged. In addition, there are also reports of other adverse reactions, such as epilepsy. Due to the small number of studies involving these adverse reactions and the relative lack of data, this paper does not carry out a detailed description and analysis.

## 5. Conclusion

The results of this meta-analysis showed that compared with craniotomy, stereotactic hematoma removal can reduce the incidence of postoperative disability, intracranial infection, pulmonary infection and gastrointestinal ulcer in middle-aged and elderly patients with hypertensive intracerebral hemorrhage, which should be interpreted carefully. Further trials are needed to prove whether the selection of types of stereotactic hematoma removal and the improvement of surgical details can further improve the surgical prognosis of middle-aged and elderly patients with hypertensive intracerebral hemorrhage.

## Author contributions

**Conceptualization:** Chao Tang.

**Data curation:** Chao Tang, Min Zhang.

**Formal analysis:** Chao Tang.

**Investigation:** Wei Li.

**Methodology:** Chao Tang, Min Zhang.

**Resources:** Wei Li.

**Software:** Min Zhang.

**Writing – original draft:** Chao Tang, Min Zhang, Wei Li.

**Writing – review & editing:** Chao Tang.

## Supplementary Material


